# The Reliability and Validity of Manual Palpation to Assess Lower Limb Arterial Occlusion Pressure in Older Adults, for Application in Blood Flow Restriction Exercise

**DOI:** 10.1002/ejsc.70233

**Published:** 2026-08-03

**Authors:** Michael Beere, Isaac Selva Raj, Jeremiah J. Peiffer, Keith D. Hill, Peter K. Edwards, Belinda Brown, Brendan R. Scott

**Affiliations:** ^1^ School of Allied Health College of Health and Education Murdoch University Perth Australia; ^2^ PHysical Activity, Sport, and Exercise (PHASE) Research Group School of Allied Health (Exercise Science) Murdoch University Perth Australia; ^3^ School of Allied Health Sciences, Faculty of Health, Discipline of Exercise and Sport Science Charles Darwin University Darwin Australia; ^4^ Rehabilitation Ageing and Independent Living (RAIL) Research Centre Monash University Peninsula Campus Frankston Australia; ^5^ School of Allied Health Curtin University Perth Australia; ^6^ Perth Orthopaedic and Sports Medicine Research Institute West Perth Australia; ^7^ Centre for Precision Health Edith Cowan University Joondalup Australia

**Keywords:** blood flow restriction, blood pressure, kaatsu training, method comparison, test‐retest reliability

## Abstract

(A) Investigate test‐retest reliability of posterior tibial pulse palpation to determine arterial occlusion pressure (AOP) and examine validity against hand‐held Doppler ultrasound. (B) Investigate the suitability of using 130% systolic brachial blood pressure for prescribing cuff inflation pressure for blood flow restriction (BFR) exercise. Repeated measures, crossover design. Lower limb AOP was assessed in 20 adults ≥ 60 years using hand‐held Doppler ultrasound and posterior tibial pulse palpation. Brachial systolic blood pressure (bSBP) was measured to predict cuff inflation pressures (bSBP_130%_). On three occasions > 24 hours apart, three AOP measurements were recorded by the same blinded rater to determine intra‐rater reliability. Participants lay supine, with arterial occlusion achieved using a rapid inflation system and a 10‐cm cuff. Reliability was assessed via intraclass correlation coefficients (ICC), coefficients of variation (CV) and Bland–Altman plots, with validity examined via Pearson's product‐moment correlations and Bland–Altman plots. Good to excellent reliability was observed for AOP assessed with Doppler ultrasound (ICC = 0.91, CV = 5.4%) and palpation (ICC = 0.91, CV = 5.8%). A strong positive correlation was observed in AOP values between methods (*r* = 0.85–0.92, *p* < 0.001), though palpation significantly underestimated AOP compared to Doppler ultrasound (−11.8 mmHg ± 8.32; *p* < 0.001). bSBP_130%_ was not different to 100% AOP determined with hand‐held Doppler (*p* = 0.516). Palpation may provide a reliable and clinically acceptable alternative to hand‐held Doppler ultrasound for assessing lower limb AOP for blood flow restriction exercise. Employing bSBP_130%_ may result in complete arterial occlusion with a 10 cm cuff, which is not supported by current BFR exercise recommendations for sub‐occlusive pressures.

## Highlights

- Palpation of the posterior tibial pulse offers a reliable and clinically acceptable alternative method for directly assessing AOP in practice, where more specialised equipment is not available.
- Palpation underestimates AOP values by −11.8 ± 8.32 mmHg compared to hand‐held Doppler ultrasound, therefore should be corrected as such.
- Employing bSBP_130%_ to prescribe cuff inflation pressure for BFR exercise may result in total arterial occlusion rather than a ‘moderate’ level of sub‐occlusive pressure when using 10 cm wide cuffs.

## 1 Introduction

Blood flow restriction (BFR) exercise relies on applying an inflatable cuff to the most proximal aspect of the upper or lower limbs, typically during low‐intensity exercise. The aim of BFR is to partially occlude arterial inflow to the working muscle group while mostly occluding venous return to the central circulation (Freitas et al. 2021). Applying BFR has demonstrated improved skeletal muscle strength and size development when combined with low‐load resistance training (Freitas et al. 2021; Miller et al. 2021), and improves cardiovascular fitness when used with low‐intensity aerobic exercise across populations (e.g., young athletes, older adults and clinical populations) (Formiga et al. 2020; Abe et al. 2010; Bennett and Slattery 2019). Originally, cuff pressure prescription during BFR exercise was arbitrary and often applied as a standardised absolute pressure regardless of individual characteristics (Abe et al. 2006; Evin et al. 2021). However, the most recent guidelines for employing BFR while at rest and during exercise highlight that individualised cuff pressure should be applied at 40%–80% of arterial occlusion pressure (AOP; the minimum pressure required to occlude arterial blood flow using the same cuffs and limbs as intended for BFR exercise) (McEwen et al. 2018; Patterson et al. 2019).

Accurate prescription of BFR cuff pressure is critical, as higher pressures can exaggerate metabolic and cardiovascular responses (Das and Paton 2022; Ilett et al. 2019; Lixandrao et al. 2015), and may increase risks of adverse events such as subcutaneous haemorrhage (bruising), muscle and nerve damage or thrombus development (Loenneke et al. 2011). To accurately assess individual AOP, equipment such as hand‐held Doppler ultrasound is required. Laurentino et al. 2020 have previously assessed the concurrent validity of hand‐held Doppler ultrasound against real‐time ultrasound for determining lower limb AOP. Although they determined hand‐held Doppler was valid, the reliability of this method was not reported. While hand‐held Doppler ultrasound is commonly employed to assess AOP in research, its inter‐session reliability is poorly reported in the literature. Nevertheless, accessibility to such equipment can be limited in clinical practice, restricting the implementation of BFR exercise or leading practitioners to prescribe suboptimal or potentially unsafe BFR cuff pressures.

Exercising with BFR is becoming increasingly popular in clinical and rehabilitation settings, including within older populations who may be load‐intolerant (Scott et al. 2024). Although low‐load BFR exercise may enable these populations to improve their physical development without vigorous training (Cahalin et al. 2022; Centner et al. 2019; Clarkson et al. 2017), a recent survey of allied health professionals identified that prescription of BFR exercise is often limited by a lack of access to necessary equipment (Scott et al. 2024). As such, developing alternative approaches to assess AOP which do not require hand‐held Doppler ultrasound is required to allow for broader implementation of BFR exercise in clinical practice. Practical alternatives for estimating AOP using pulse oximetry have been examined, with inconsistent findings (Zeng et al. 2019; Brekke et al. 2020). Equations to estimate AOP have also been developed (Loenneke et al. 2015); however, they do not account for differences in equipment (e.g., cuff width or material), individual characteristics or body position during assessment, and therefore have limited applicability beyond the specific context in which they were established. One example is the use of 130% brachial systolic blood pressure (bSBP_130%_), which, when applied to the lower limb, is suggested to deliver a ‘moderate’ pressure stimulus (Suga et al. 2010). This method has been used as a surrogate to prescribe cuff inflation pressure when equipment for directly assessing lower limb AOP may not be available (Doma et al. 2020; de Queiros et al. 2025). However, a recent study found that this particular method may in fact completely occlude blood flow when using a wide (18 cm) cuff (de Queiros et al. 2025). Palpation of a peripheral pulse may provide an alternative, having been previously investigated to clinically screen for ankle‐brachial index (ABI) by palpating the posterior tibial pulse (Aboyans et al. 2012). When identifying the posterior tibial pulse, palpation demonstrated a specificity of 88% and sensitivity of 72%–82% compared to hand‐held Doppler ultrasound, in an older population (64.5 years) (Aboyans et al. 2008; Migliacci et al. 2008). These outcomes were deemed acceptable for palpation to be used as a pragmatic method for General Practitioners to assess ABI when screening for peripheral arterial disease, when ultrasound is not available.

To our knowledge, there has been no investigation of using manual palpation to assess lower limb AOP for the purpose of prescribing individualised cuff pressure for BFR exercise. Therefore, the primary aim of this study was to determine the reliability and validity of palpating the posterior tibial pulse to assess lower limb AOP, compared to established techniques using hand‐held Doppler ultrasound. A secondary aim was to investigate the suitability of a commonly employed method, bSBP_130%_ for prescribing cuff inflation pressure for BFR exercise. To ensure the outcomes were transferable to populations who may benefit from BFR exercise, these aims were examined in a sample of older adults without contraindications to BFR. Our hypotheses were: (1) palpation of the posterior tibial pulse will demonstrate at minimum good intra‐rater reliability for assessing AOP in adults > 60 years, (2) palpation will demonstrate clinically acceptable agreement, previously defined as falling within ± 15 mmHg of the group mean (Hughes and McEwen 2021) with hand‐held Doppler ultrasound when assessing lower limb AOP and (3) bSBP_130%_ will not be suitable for prescribing cuff inflation pressure.

## 2 Methods

### 2.1 Participants

Twenty healthy male (*n* = 6) and female (*n* = 14) participants over the age of 60 participated in the current study. An a priori sample size estimate was conducted via the hypothesis testing approach and online calculator provided by Borg et al. 2022. The minimum acceptable intraclass correlation coefficient (ICC) was set at 0.70, with an expected ICC of 0.90 based on three assessments per participant (Karanasios et al. 2022). Hence, a sample size of 17 participants (power = 0.80, *p* = 0.05) was considered adequate, with 20 participants recruited to account for 15% attrition. Potential participants were contacted via email or telephone and provided an overview of the study purpose, methods and potential risks. They were then assessed for initial eligibility and excluded if they self‐reported: uncontrolled hypertension, neuromuscular disease, terminal disease, myocardial infarction in the past 6 months, unstable cardiovascular disease or a lower limb fracture within the last 6 months. A BFR‐specific screening questionnaire was completed at the first session prior to commencing the assessments (Kacin et al. 2015), and medical clearance was required if they presented with any of the relative exclusion criteria. Prior to commencing the study, participants provided signed informed consent to proceed. The study was approved by the Murdoch University Human Research Ethics Committee (project number 2024/009).

### 2.2 Experimental Design

Participants attended the laboratory for three assessment sessions on separate days with no less than 24 hours between sessions. Where possible, participants attended subsequent sessions at the same time of day, or at least in the morning (before midday) or afternoon (after midday), to limit diurnal fluctuations in blood pressure (Ingram et al. 2017). They were asked to avoid caffeine, alcohol and physical activity 12 hours prior to the assessment, with confirmation provided by the participant on the assessment day. Participants' height, body mass and resting (seated) brachial blood pressure were recorded before every session. Thigh girth of the limb being assessed for AOP (cm; 50% of the distance between the greater trochanter and lateral femoral condyle) (Hughes et al. 2018) was recorded during the first session only. This was followed by assessment of lower limb AOP in a supine position. On the first assessment day, both the limb to be assessed (left or right) and first measurement method (hand‐held Doppler ultrasound or palpation) were randomised, determined by one researcher drawing each selection from an envelope. Within each session, the method (hand‐held Doppler ultrasound or palpation) was alternated for each subsequent AOP measurement. Across sessions, the same alternating order of measurement method was used on the same limb. Randomisation was counterbalanced, ensuring an equal number of participants began with each measurement method. A rapid inflation system (Hokanson E20 Rapid Cuff Inflator with AG101 air source, Washington, USA) with nylon cuff and polyurethane bladder (SC10D, 10 cm wide bladder, 85 cm long) was used to occlude lower limb blood flow. A Hokanson MD6 bidirectional hand‐held Doppler ultrasound was used to identify the posterior tibial pulse. Complete details of the inflation device, tourniquet characteristics and hand‐held Doppler are provided in Table S1. A total of six measurements were taken each session: three measurements with hand‐held Doppler ultrasound, identifying the posterior tibial pulse, and three measurements via manual palpation of the posterior tibial pulse. If the tibial pulse was not manually palpable, the dorsalis pedis pulse was used as an alternative (1 out of 20 participants). Each AOP measurement took between 30 and 60 seconds to complete (including initially locating the posterior tibial pulse prior to cuff inflation). The researcher operating the inflation device and identifying the pulse was blinded to the AOP measurement (the numerical display on the inflation device was covered during inflation and deflation), which was then uncovered and recorded by a second researcher once AOP had been determined. The second and third assessment sessions followed the same procedure as the first.

### 2.3 Assessing Arterial Occlusion Pressure

For all AOP assessments, participants removed both shoes and socks to allow clear access to the posterior tibial pulse. The cuff was placed around their proximal thigh on the side to be assessed. The top edge of the cuff was positioned as close as possible to the gluteal fold, tight enough that it did not slide down while standing, and the cuff bladder was positioned medially to ensure pressure was applied directly over the femoral artery. The cuff position was recorded, measuring the distance from the distal edge of the cuff to the superior pole of the patella while supine to allow for accurate, repeated placement in subsequent sessions. Participants lay in a supine position with both arms by their sides for five minutes prior to the first AOP measurement. A continuous inflation method was employed to achieve complete arterial occlusion: the cuff was gradually and continuously inflated until a pulse was no longer detectable (∼15–20 seconds); it was then gradually and continuously deflated (∼5–10 seconds) (Vehrs et al. 2023). AOP was defined as the point at which the pulse was detected again, and recorded to the nearest 1 mmHg. Once AOP was determined, the cuff was immediately and completely deflated. Participants then rested in supine for five minutes between each measurement.

Using the hand‐held Doppler ultrasound, the probe was applied with ample conductive ultrasound gel and placed on the posterior tibial pulse until a clear pulse (auscultatory signal) was detected as per manufacturer directions. The cuff was then inflated from 0 mmHg until complete arterial occlusion was reached. The palpation assessment method followed the same process as with hand‐held Doppler ultrasound, but the researcher manually palpated the posterior tibial pulse with their index and middle fingers to determine the point of complete blood flow occlusion. Three AOP measurements were taken using each measurement method during each session.

### 2.4 Determination of Cuff Inflation Pressure With bSBP_130%_

Brachial blood pressure was measured at the start of each testing session. This was assessed with an automatic blood pressure monitor (HEM‐7120; OMRON Healthcare, Singapore) and standard 18 cm wide single chamber nylon cuff after participants had been resting in a seated position for at least five minutes. Two readings were taken, and the mean bSBP from each session was used to calculate bSBP_130%_ (bSBP × 1.3) as reported in previous research (Doma et al. 2020; de Queiros et al. 2025; Loenneke et al. 2013).

### 2.5 Statistical Analysis

Data were analyzed using the mean of the three AOP measurements taken during each testing session, for each method. Shapiro–Wilk test was used to assess statistical normality of the data along with visual inspection of histograms and Q‐Q plots. One‐way repeated measures ANOVA was performed to determine mean differences among sessions for each method. A 2 × 3 (method vs. session) repeated measures ANOVA with Bonferroni correction for multiple comparisons was performed to determine mean differences between Doppler and palpation methods, and between Doppler and bSBP_130%_. Post‐hoc *t‐*tests were conducted if a significant interaction effect, or main effect of method or session was observed. Intra‐rater reliability for all three measurement methods was assessed with a 2‐way mixed‐effects, absolute agreement ICC_(2,*k*)_ model with 95% confidence intervals (CI) and calculated based on a mean rating (Koo and Li 2016). To examine the absolute variability of each method across sessions, the coefficient of variation (CV) was calculated as (SD ÷ mean) × 100 using the combined session mean and SD of each method. The ICC's 95% CIs with the following ratings: poor = < 0.5, moderate = 0.5–0.75, good = 0.75–0.9, excellent ≥ 0.9^36^ and CV were used to provide a rating of reliability. Standard error of measurement (SEM) was used to determine absolute intra‐rater reliability and calculated as SD × √(1‐ICC). Minimal detectable change (MDC) was also calculated with 95% confidence (SEM × 1.96 × √2) to determine a minimum threshold for measurement error. Cohen's dz was calculated (*t* ÷ √n) to determine the within‐subjects effect for each testing session and categorised as small (0.2), medium (0.5) and large (0.8) (Lakens 2013). Bland–Altman plots with 95% limits of agreement (± 1.96 × standard deviation) were used to visually assess the agreement between Doppler and palpation, and between Doppler and bSBP_130%_, and within each method between different testing sessions (Session 1 vs. Session 2, Session 1 vs. Session 3 and Session 2 vs. Session 3). Outliers were not removed from the Bland–Altman analyses as it is suggested these analyses are not overly sensitive to a small number of large outliers (Bland and Altman 1999). Deviation in cuff pressure of ± 15 mmHg has previously been defined as a clinically acceptable range as it is often used in surgical tourniquet systems when maintaining a blood‐free field (Hughes and McEwen 2021). Therefore, in the current study, a ± 15 mmHg difference in AOP from the group mean, was considered a clinically acceptable range. Pearson's product‐moment correlation coefficients (*r*) were calculated using session means to assess the relationship of AOP values measured via palpation compared to hand‐held Doppler and bSBP_130%_. All statistical analyses were conducted with SPSS software version 30 (SPSS Inc., Chicago, IL), with the alpha level set at *p* ≤ 0.05. Unless indicated otherwise, all data are shown as mean ± standard deviation.

## 3 Results

A total of 20 participants aged 60–84 years (71.5 ± 5.31 years) completed the current study with no dropouts, and no participants were excluded after baseline screening. Detailed participant characteristics are presented in Table 1.

**TABLE 1:** Participant characteristics at the first testing session.

Sex	Males	Females	Total
	*N* = 6	*N* = 14	*N* = 20
Age (years)	72.6 ± 9.9	71.1 ± 4.9	71.5 ± 5.3
Height (cm)	175.6 ± 6.1	164.1 ± 9.2	167.6 ± 9.8
Body mass (kg)	83.0 ± 7.4	70.2 ± 9.1	74.1 ± 10.4
SBP (mmHg)	140.3 ± 14.1	132.1 ± 18.5	134.5 ± 17.4
DBP (mmHg)	81.6 ± 4.8	74.1 ± 11.1	76.4 ± 10.1
Thigh girth (cm)	51.2 ± 2.8	50.2 ± 3.6	50.5 ± 3.3

*Note:* All values presented as mean ± SD.

Abbreviations: DBP, diastolic blood pressure; SBP, systolic blood pressure.

### 3.1 Test‐Retest Reliability of Arterial Occlusion Pressure

**FIGURE 1:**
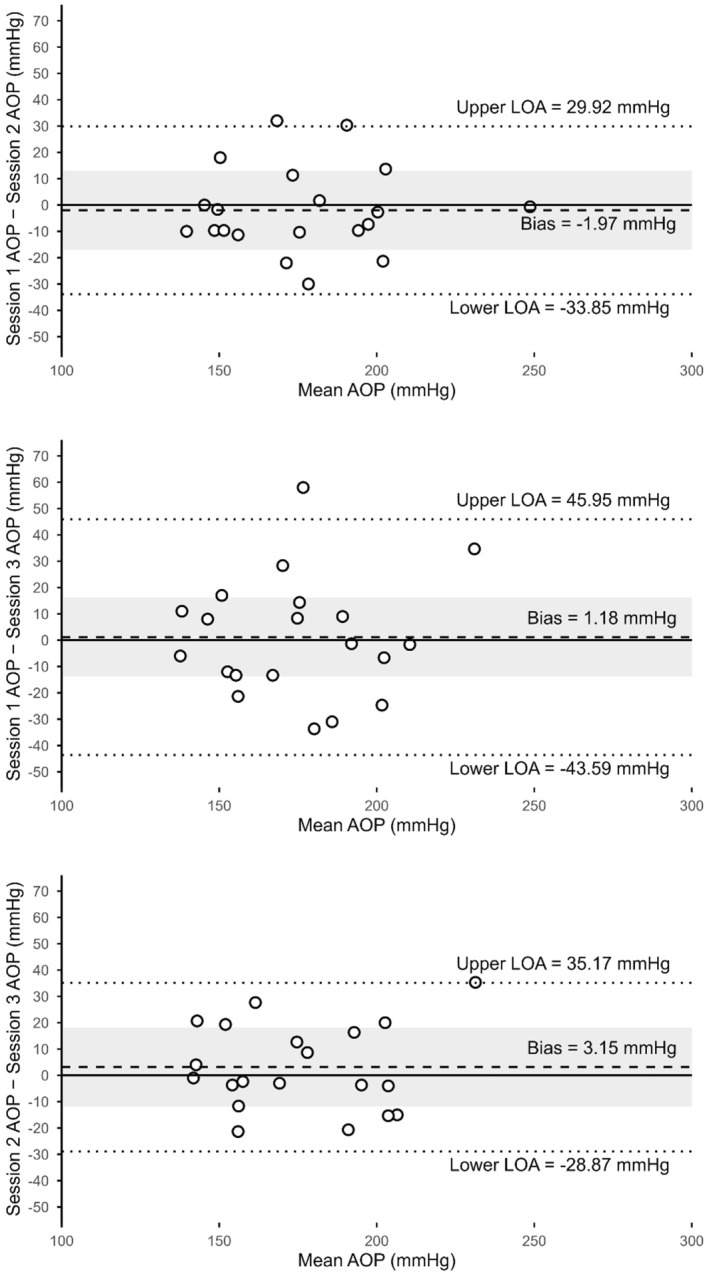
Bland–Altman plots of the agreement in AOP values taken with hand‐held Doppler ultrasound between testing sessions. Top panel = Sessions 1 versus 2; middle panel = Sessions 1 versus 3; bottom panel = Sessions 2 versus 3. Shaded area denotes ± 15 mmHg around the group mean. AOP: Arterial occlusion pressure; LOA: Limits of Agreement.

The mean AOP values obtained via hand‐held Doppler ultrasound did not differ among sessions one (175.3 ± 28.4 mmHg), two (177.3 ± 27.9 mmHg) and three (174.1 ± 26.8 mmHg), *F*(2,38) = 0.288, *p* = 0.751. The ICC and CV for hand‐held Doppler are presented in Table 2, together indicating good to excellent intra‐rater reliability (Laurentino et al. 2020; Koo and Li 2016). Absolute reliability was further demonstrated with relatively low SEM (Brekke et al. 2020) across testing sessions; however, MDC values fell outside the clinically acceptable range of ± 15 mmHg from the mean difference (Table 2). Relatively wide limits of agreement were observed for AOP values taken via hand‐held Doppler ultrasound between sessions (Figure 1). However, 65% (Sessions 2 and 3) and 70% (Session 1) of AOP values (13 and 14 out of 20, respectively) were within a clinically acceptable range of ± 15 mmHg from the mean difference, for cuff pressure variations (Hughes and McEwen 2021).

**TABLE 2:** Intrarater reliability for hand‐held Doppler ultrasound and palpation.

	ICC [95% CI]	CV (%)	SEM (mmHg)	MDC (mmHg)
Hand‐held Doppler	0.91 [0.81–0.96]	5.4	7.64	21.18
Palpation	0.91 [0.82–0.96]	5.8	8.14	22.56
bSBP_130%_	0.94 [0.87–0.97]	4.7	6.82	18.92

Abbreviations: CV, coefficient of variation; ICC, Intraclass correlation coefficient; MDC, Minimal detectable change; mmHg, millimetres of mercury; SEM, standard error of measurement.

**FIGURE 2:**
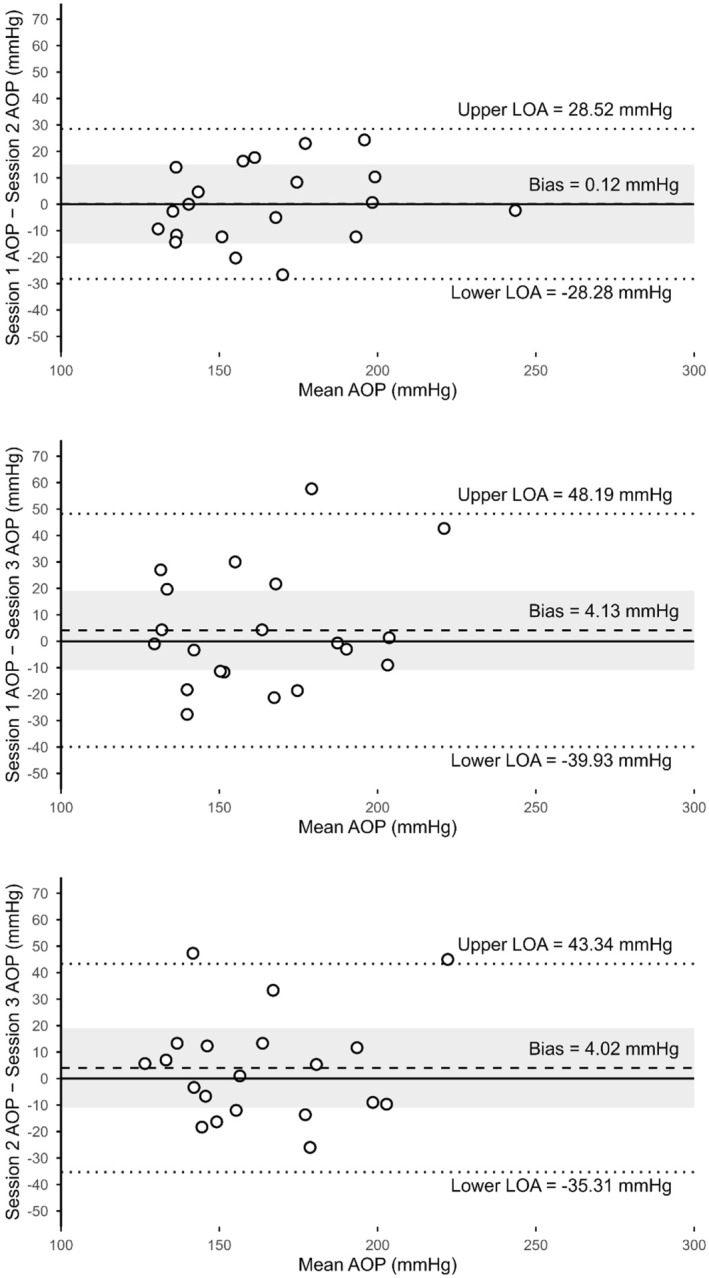
Bland–Altman plots of the agreement in AOP values taken with Palpation between testing sessions. Top panel = Sessions 1 versus 2; middle panel = Sessions 1 versus 3; bottom panel = Sessions 2 versus 3. Shaded area denotes ± 15 mmHg around the group mean. AOP, Arterial occlusion pressure; LOA, Limits of Agreement.

Mean AOP measurements taken via palpation over the 3 testing sessions were 165.20 ± 31.67 mmHg, 165.08 ± 28.82 mmHg and 161.06 ± 27.33 mmHg, respectively, and were not significantly different (*F*(2,38) = 0.595, *p* = 0.557). Palpation demonstrated good‐to‐excellent reliability as indicated by ICC and CV values (Laurentino et al. 2020) in Table 1. Reliability is further demonstrated by the relatively low SEM (Brekke et al. 2020); however, MDC values extend beyond ± 15 mmHg from the mean difference (Table 1). Bland–Altman plots again demonstrate wide limits of agreement between sessions (Figure 2), with 70% (Sessions 1 and 2) and 50% (Session 3) of AOP measurements falling ≤ ± 15 mmHg from the mean difference (Figure 2).

### 3.2 Agreement Between Methods of Measuring Arterial Occlusion Pressure

**FIGURE 3:**
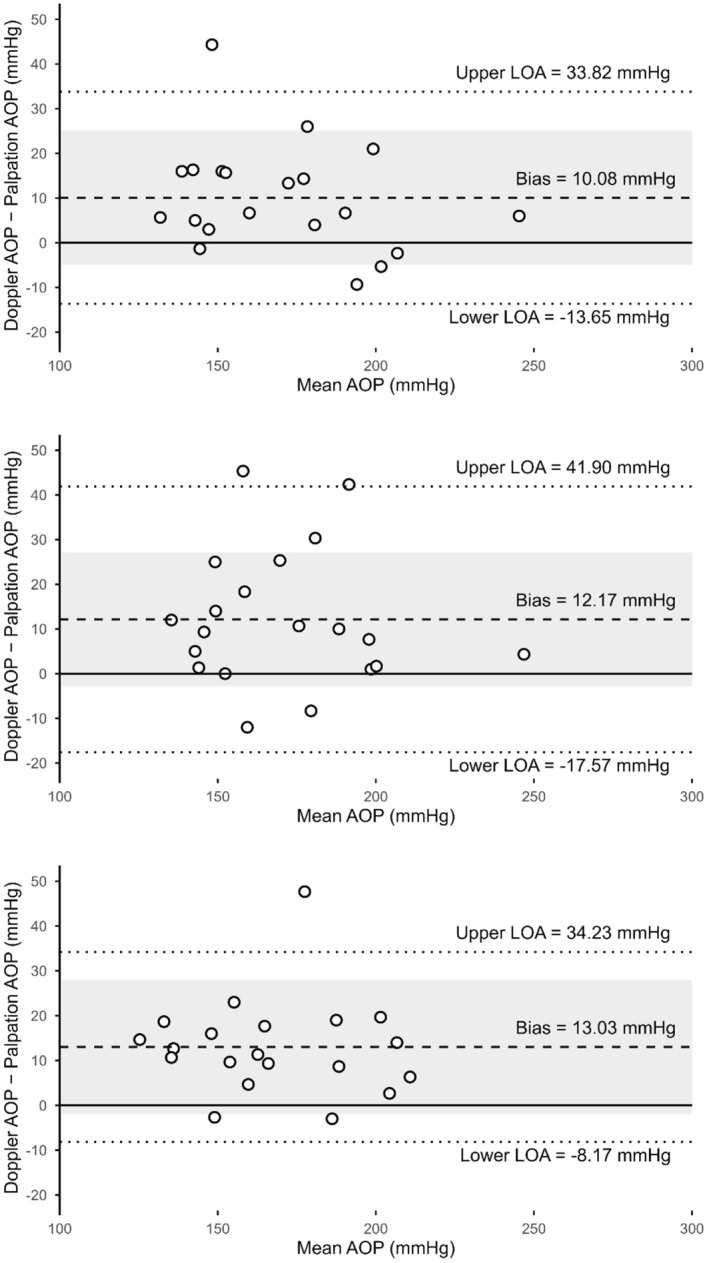
Bland–Altman plots of the agreement in AOP between hand‐held Doppler ultrasound and palpation methods between testing sessions. Top panel = Session 1; middle panel = Session 2; bottom panel = Session 3. The shaded area denotes ± 15 mmHg around the group mean. AOP, Arterial occlusion pressure; LOA, Limits of Agreement.

**FIGURE 4:**
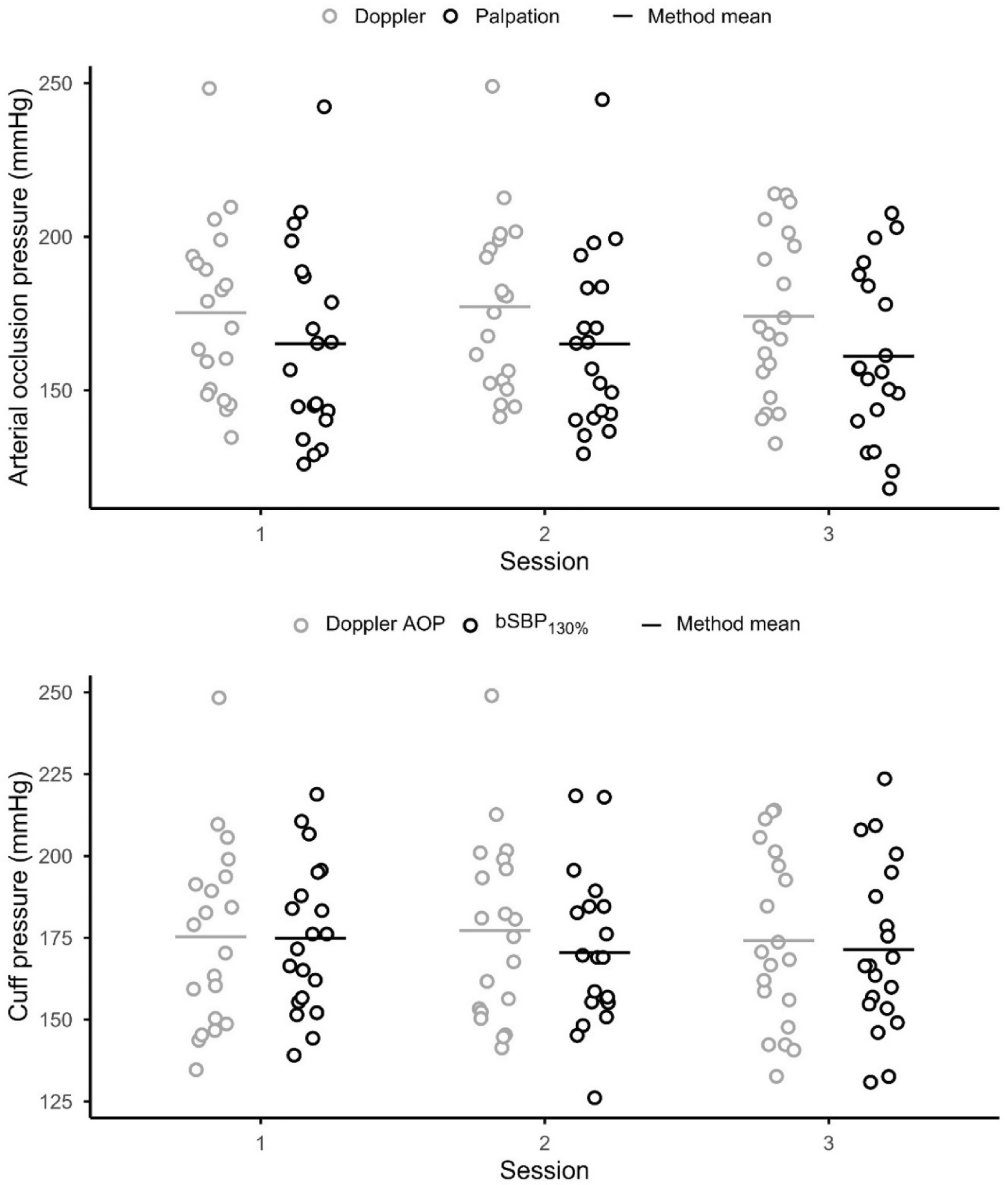
Comparison of individual AOP values per testing session. Top panel = AOP assessed with Doppler ultrasound and palpation. Bottom panel = AOP assessed with Doppler ultrasound and 130% brachial systolic blood pressure (130%SBP).

There was a significant effect for measurement method whereby hand‐held Doppler ultrasound AOP values were significantly higher than palpation (*F*(1,19) = 21.40, *p* < 0.001) across all sessions (10.1 ± 7.13 mmHg, 12.2 ± 8.60 mmHg and 13.0 ± 9.21 mmHg for Sessions 1, 2 and 3, respectively). Additionally, the effect size for the method was determined as large for all sessions (Table 3). Bland–Altman plots (Figure 3) show a wide range between the upper and lower limits of agreement across testing sessions one (−13.7 to +33.8 mmHg), two (−17.6 to +41.9 mmHg) and three (−8.2 to +34.2 mmHg) when comparing measurement methods, demonstrating a moderate level of agreement between AOP values obtained via each method. Nevertheless, between 75% (Doppler ultrasound, 14 of 20) and 65% (palpation, 13 of 20) of observations fell within a clinically acceptable range of ± 15 mmHg of the mean difference (Hughes and McEwen 2021). Furthermore, Pearson's correlation showed strong positive correlations between the hand‐held Doppler ultrasound and palpation methods for Session 1 (*r* = 0.92 [CI = 0.81–0.97], *p* < 0.001), Session 2 (*r* = 0.86 [CI = 0.67–0.94], *p* < 0.001) and Session 3 (*r* = 0.92 [CI = 0.80–0.97], *p* < 0.001). The spread and group mean of individual AOP values as determined with Doppler ultrasound and palpation for each testing session are presented in Figure 4.

**TABLE 3:** Session means for arterial occlusion pressure by method and session.

				Doppler versus palpation		Doppler versus bSBP130%	
Session	Doppler	Palpation	bSBP130%	*t*	*d*_*z*_	*t*	*d*_*z*_
1	175.3 ± 28.4	165.2 ± 31.7	174.9 ± 22.6	3.72**	0.82	0.55	0.12
2	177.3 ± 27.9	165.1 ± 28.8	170.5 ± 23.6	3.58**	0.80	1.13	0.25
3	174.1 ± 26.7	161.1 ± 27.3	171.3 ± 25.7	5.38***	1.20	0.64	0.14

*Note:* All pressure values are presented as mean ± SD (mmHg).

^**^ *p* ≤ 0.002.

^***^ *p* < 0.001.

### 3.3 130% Brachial Systolic Blood Pressure Compared to Doppler Ultrasound

**FIGURE 5:**
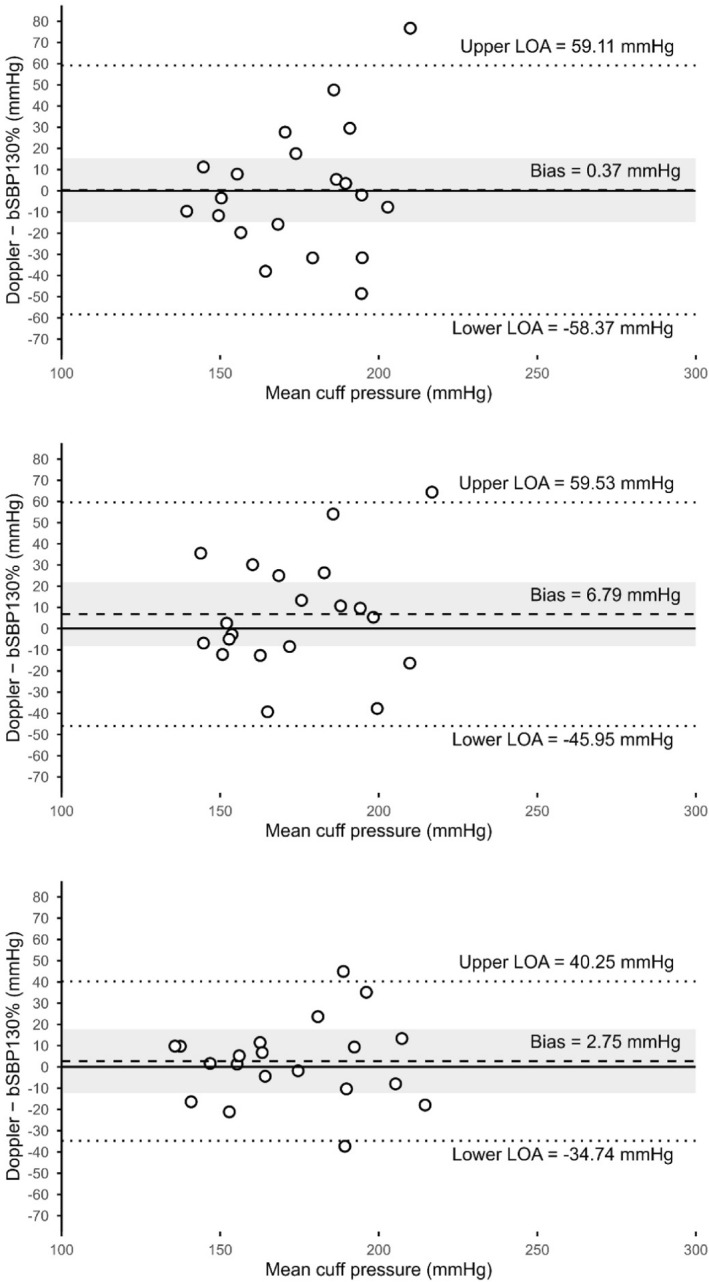
Bland–Altman plots of the agreement in cuff pressure values between hand‐held Doppler ultrasound and bSBP_130%_ methods between testing sessions. Top panel = Session 1; middle panel = Session 2; bottom panel = Session 3. The shaded area denotes ± 15 mmHg around the group mean. LOA, Limits of Agreement.

There was no difference in cuff pressures reported between Doppler ultrasound‐measured AOP and cuff pressure derived from bSBP_130%_ (*F*(2,38) = 0.439, *p* = 0.516). Mean cuff pressure values per session for these methods are presented in Table 3. Bland–Altman plots (Figure 5.) show a wide range between the upper and lower limits of agreement across testing sessions one (−58.37 to +59.11 mmHg), two (−45.95 to +59.53 mmHg) and three (−34.74 to +40.25 mmHg) when comparing measurement methods, demonstrating poor agreement between cuff pressure values obtained via each method. No correlation was observed between the Doppler ultrasound and bSBP_130%_ methods for Session 1 (*r* = 0.33 [CI = −0.136–0.67], *p* = 0.161), however, moderate positive correlations were observed for Session 2 (*r* = 0.46 [CI = 0.027–0.752], *p* = 0.039) and Session 3 (*r* = 0.74 [CI = 0.44–0.89], *p* < 0.001). Further, bSBP_130%_ exceeded Doppler‐derived AOP in 11 (55%), 11 (55%) and 10 (50%) of individuals in sessions one, two and three, respectively. The spread and group mean of individual cuff pressure values as determined with Doppler ultrasound and bSBP_130%_ for each testing session are presented in Figure 4.

## 4 Discussion

The primary aims of the present study were as follows: (1) to investigate the validity and reliability of palpation of the posterior tibial pulse to assess lower limb AOP in adults ≥ 60 years, and (2) to determine whether the agreement between palpation and hand‐held Doppler ultrasound was within a previously defined clinically acceptable range (± 15 mmHg of the group mean) (Hughes and McEwen 2021). The secondary aim of this study was to investigate the suitability of employing bSBP_130%_ to prescribe ‘moderate’ sub‐occlusive cuff inflation pressure for BFR exercise. The main findings of the current study were: (1) palpation demonstrated good‐to‐excellent test‐retest reliability for assessing lower limb AOP, which was comparable to hand‐held Doppler ultrasound, but systematically underestimated AOP values, (2) the agreement between AOP values obtained via palpation and hand‐held Doppler ultrasound was clinically acceptable. The secondary findings indicated that cuff pressure values based on bSBP_130%_ were not different to 100% AOP values determined with Doppler ultrasound, indicating that bSBP_130%_ may not be a suitable option for prescribing sub‐occlusive cuff pressures during BFR exercise. Taken together, these findings support our hypotheses and suggest that palpation may provide a reliable and valid alternative to hand‐held Doppler ultrasound for assessing AOP. Further, assessing AOP via manual palpation is likely more suitable for subsequent BFR cuff pressure prescription than using bSBP_130%_.

### 4.1 Test‐Retest Reliability of AOP Assessment Methods

In the current study, the test‐retest reliability of palpation of the posterior tibial pulse was comparable to that of the hand‐held Doppler ultrasound. These results align with a recent study by Chesbro et al. (2011), who reported the same hand‐held Doppler ultrasound device had very strong test‐retest reliability for identifying the posterior tibial pulse in a supine position (ICC = 0.93–0.99 [95% CI = 0.81–0.99]). Palpation of the posterior tibial pulse has previously demonstrated high levels of specificity and sensitivity, 88% and 82%, respectively, when assessing ABI compared to hand‐held Doppler ultrasound (Migliacci et al. 2008). However, in these studies and in contrast to the current results, palpation demonstrated poor reproducibility (intra‐observer R‐coefficient = 0.61 [*p* < 0.05]; CV 23%) when compared to Doppler ultrasound (Aboyans et al. 2012, 2008). These outcomes may be symptomatic of the clinical population in the aforementioned studies (patients with peripheral and central cardiac conditions impacting peripheral vascular function), considering that posterior tibial pulse was not detected by palpation in 43 of 54 participants (Aboyans et al. 2008), and in 9 of 205 participants (Migliacci et al. 2008). In the current study, posterior tibial pulse was not detected with palpation in only one participant, though it was detectable with Doppler ultrasound. In this instance the dorsalis pedis pulse was used for both assessment methods and the participant's AOP values were within the group trends. Inspection of Bland–Altman plots reveals similarly wide limits of agreement between sessions for both Doppler ultrasound and palpation methods. This may reflect inter‐session individual and methodological differences within both measurement methods. Although the limits of agreement observed for the Doppler ultrasound were larger than expected for a validated reference method, this is consistent with previous research where lower limb vascular measurements demonstrate inter‐day variability (Daniele et al. 2024). Furthermore, although the Doppler method has been previously validated (Laurentino et al. 2020) and demonstrated strong inter‐ and intrarater reliability (Chesbro et al. 2011), these studies were performed in a single session. There has been no prior rigorous examination of hand‐held Doppler to measure lower limb AOP across time points; therefore, comparison to previous literature is limited. When considering both SEM and MDC values in the current results, there seems to be a moderate level of variability within each method of measurement from session to session, some of which is likely due to differences between participants (Evin et al. 2021). Nevertheless, most AOP readings were within an acceptable range for both measurement methods.

### 4.2 The Validity of Palpation to Assess Lower Limb AOP

Hand‐held Doppler ultrasound has previously been validated for assessing lower limb AOP against real‐time ultrasound, which is considered the gold standard (Laurentino et al. 2020). In the current study, AOP values determined via palpation displayed a strong, positive and linear relationship to those of hand‐held Doppler ultrasound. However, AOP values reported with the hand‐held Doppler ultrasound were significantly higher across all sessions compared to palpation in the current study, suggesting a systematic difference. Similar findings were reported by Zeng et al. 2019, demonstrating higher AOP values determined with hand‐held Doppler ultrasound compared to pulse oximetry. In both cases, this may be explained by the greater sensitivity of the Doppler ultrasound for detecting pulse, by way of sound rather than infrared (pulse oximetry) or feeling for arterial deformation through layers of tissue (palpation). Pulse pressure has previously been identified as strongly associated with AOP (Evin et al. 2021) and may contribute to measurement accuracy when employing a method such as palpation; however, this was not investigated in the current study. Although significant differences were observed between AOP measurement methods (Doppler ultrasound and palpation) in the current study, this does not necessarily reflect poor agreement. In contrast to the study by Zeng et al. 2019, AOP measurement methods in the current study demonstrated better agreement, as indicated by the narrower limits of agreement across testing sessions. Although the LOA in the current study are narrower, it is important to note there was variability in AOP values across sessions regardless of method. As there are no previous studies that report AOP values determined via palpation compared to a gold standard, direct comparisons with the current findings are not possible (Aboyans et al. 2012). Although palpation has previously been investigated for assessing ABI, these studies did not report specific cuff pressure values, only ABI indices calculated as the brachial bSBP ÷ ankle SBP (Aboyans et al. 2008; Migliacci et al. 2008). Nevertheless, in both studies, palpation was reported to underestimate ABI indices and had a higher rate of false positive outcomes compared to Doppler ultrasound. This may suggest the AOP value measured with palpation was indeed lower.

### 4.3 Prescribing Cuff Inflation Pressure Using bSBP_130%_

Prescription of cuff inflation pressure for BFR exercise using bSBP_130%_ has been a practical option where equipment such as hand‐held Doppler ultrasound is not available. Although this method has been commonly employed in research (de Queiros et al. 2025), strong justification for its use is lacking in the literature. A recent paper by (de Queiros et al. 2025) investigated this method and found that bSBP_130%_ (seated) exceeded lower limb AOP ranging from 101% to 158% of the assessed AOP value (in supine with a hand‐held Doppler ultrasound) in a young (≤ 40 years) healthy cohort. These results align with those in the current study, where there was no difference was observed between bSBP_130%_ and the lower limb AOP values assessed via hand‐held Doppler ultrasound. However, as AOP in our study was assessed using a narrower cuff (10 cm bladder) compared to the 18 cm cuff used for bSBP, higher pressures are expected (Loenneke et al. 2012); if the same 18 cm wide cuff was used to assess AOP, it is likely bSBP_130%_ values would have also exceeded AOP values. Furthermore, the same method was employed using narrow (5 cm) and wide (13.5 cm) cuffs in a study by Loenneke et al. (2013) finding that when bSBP_130%_ was applied using the narrow cuff, 1 out of 83 (1.2%) participants reached total occlusion, whereas 49 out of 119 (41%) participants reached occlusion with the wider cuff. Again, this aligns with the current results where 50%–55% of participant's cuff pressure values determined with bSBP_130%_ exceeded Doppler‐derived AOP values across the three testing sessions. These findings highlight the importance of considering cuff width on the occlusive stimulus being delivered when using fixed pressure values. Although bSBP_130%_ may provide some degree of personalisation, its applicability is likely dependent on factors such as cuff width (Loenneke et al. 2013) and body position during assessment (supine, seated or standing) (de Queiros et al. 2025).

While this study presents novel findings regarding the use of palpation to assess lower limb AOP, it is important to acknowledge some limitations. Although all efforts were made to reduce bias where possible, repeating the same alternating AOP measurement order across each testing session may result in a learning effect for the assessor. Our sample was restricted to healthy adults aged over 60 years, which may limit generalisability to broader populations such as younger adults, athletes and clinical populations. However, BFR exercise has consistently demonstrated positive outcomes within older populations, highlighting its potential for practical application in this demographic. Furthermore, all AOP assessments in this study were conducted with laboratory‐grade tourniquets and inflation tools, and it is possible that using a manually inflatable cuff with a manual sphygmomanometer may increase the difficulty of palpation assessments by introducing additional movement by the assessor inflating the cuff. Brachial systolic blood pressure was assessed in a seated position, potentially leading to higher cuff pressures estimated by bSBP_130%_ than if it were assessed in supine, as AOP was with Doppler ultrasound. Finally, the outcomes presented in the current study are specific to its context and methods (e.g., cuff width and material, body position), and further investigation is needed to examine manual palpation of AOP under other conditions and in a wider population.

## 5 Conclusion

Equipment‐based methods of assessing AOP such as hand‐held Doppler remain the preferred method for research and clinical practice, however, palpation of the posterior tibial pulse is a reliable and clinically acceptable alternative for assessing lower limb AOP in populations ≥ 60 years while using 10 cm wide cuffs. Although the current results indicate that at a group mean level palpation underestimates AOP compared to Doppler ultrasound, this difference may be accounted for by adding 11.8 mmHg to the palpated AOP value. Nevertheless, practitioners should still exercise caution if taking this approach, as variability was observed in all AOP assessment methods. Palpation seems to provide a pragmatic and direct AOP assessment method which accounts for primary determinants of AOP. Employing bSBP_130%_ to prescribe cuff pressure should also be done so with caution, as it may in fact cause total occlusion of the limb rather than only partial occlusion.

## Funding

The first author is supported by an Australian Government Research Training Program scholarship. The last author is supported by an NHMRC Investigator Grant (GNT1196462).

## Conflicts of Interest

The authors declare no conflicts of interest.

## Supporting information


**Table S1:** Inflation device, tourniquet characteristics and occlusion pressure assessment methodology.

## Data Availability

The data that support the findings of this study are available on request from the corresponding author. The data are not publicly available due to privacy or ethical restrictions.
