# Supplementary material for: The Reliability and Validity of Manual Palpation to Assess Lower Limb Arterial Occlusion Pressure in Older Adults, for Application in Blood Flow Restriction Exercise
Source: Eur J Sport Sci. 2026 Aug 3;26(8):e70233. doi: 10.1002/ejsc.70233 (PMC13434030; doi:10.1002/ejsc.70233)
Supplement: Supplementary file 1 [file EJSC-26-e70233-s001.docx]

**Supplementary Table 1**. Inflation device, tourniquet characteristics and occlusion pressure assessment methodology

| **BFR inflation device** | |
| --- | --- |
| **Manufacturer and model** | Hokanson E20 Rapid Cuff Inflator with AG101 air source |
| **Method of pressure delivery** | Automated and manual |
| **Validation of device** | Reference standard^1^ |
| **Tourniquet properties** | |
| **Manufacturer and model** | Hokanson SC10D™ |
| **Cuff dimensions (external)** | Width: 11.5cm  Length: 83.5cm |
| **Internal bladder dimensions** | Width: 10cm  Length: 40.5cm; does not fully encompass limb |
| **Material** | Nylon cuff with polyethylene bladder |
| **Type of internal bladder system** | Single chambered |
| **Cuff Shape** | Straight cuff |
| **AOP measurement** | |
| **Arterial occlusion pressure** | Absolute pressure required to completely occlude blood flow to the limb, in mmHg. |
| **Posture used for measurement of AOP** | Supine |
| **Inflation method** | Continuous |
| **Blood flow detection method** | Blood flow detected at the posterior tibial pulse using:   - Hokanson MD6 bidirectional hand-held Doppler ultrasound (validated)^2^ - Manual palpation of posterior tibial pulse (experimental method) |

BFR, blood flow restriction. AOP, arterial occlusion pressure

1.	Loenneke JP, Fahs CA, Rossow LM, et al. Effects of cuff width on arterial occlusion: implications for blood flow restricted exercise. *Eur J Appl Physiol.* 2012;112(8):2903-2912.

2.	Laurentino GC, Loenneke JP, Mouser JG, et al. Validity of the Handheld Doppler to Determine Lower-Limb Blood Flow Restriction Pressure for Exercise Protocols. *J Strength Cond Res.* 2020;34(9):2693-2696.
